# A Novel COVID-19 Diagnosis Approach Utilizing a Comprehensive Set of Diagnostic Information (CSDI)

**DOI:** 10.3390/jcm12216912

**Published:** 2023-11-03

**Authors:** Ulzhalgas Zhunissova, Róża Dzierżak, Zbigniew Omiotek, Volodymyr Lytvynenko

**Affiliations:** 1 Department of Biostatistics, Bioinformatics and Information Technologies, Astana Medical University, Beibitshilik Street 49A, Astana 010000, Kazakhstan; 2 Faculty of Electrical Engineering and Computer Science, Lublin University of Technology, Nadbystrzycka 38 A, 20-618 Lublin, Poland; 3 Department of Informatics and Computer Science, Kherson National Technical University, Beryslavs’ke Hwy, 24, 730082 Kherson, Kherson Oblast, Ukraine

**Keywords:** COVID-19, machine learning, data preprocessing, feature selection, classification

## Abstract

The aim of the study was to develop a computerized method for distinguishing COVID-19-affected cases from cases of pneumonia. This task continues to be a real challenge in the practice of diagnosing COVID-19 disease. In the study, a new approach was proposed, using a comprehensive set of diagnostic information (CSDI) including, among other things, medical history, demographic data, signs and symptoms of the disease, and laboratory results. These data have the advantage of being much more reliable compared with data based on a single source of information, such as radiological imaging. On this basis, a comprehensive process of building predictive models was carried out, including such steps as data preprocessing, feature selection, training, and evaluation of classification models. During the study, 9 different methods for feature selection were used, while the grid search method and 12 popular classification algorithms were employed to build classification models. The most effective model achieved a classification accuracy (ACC) of 85%, a sensitivity (TPR) equal to 83%, and a specificity (TNR) of 88%. The model was built using the random forest method with 15 features selected using the recursive feature elimination selection method. The results provide an opportunity to build a computer system to assist the physician in the diagnosis of the COVID-19 disease.

## 1. Introduction

**Figure 1:**
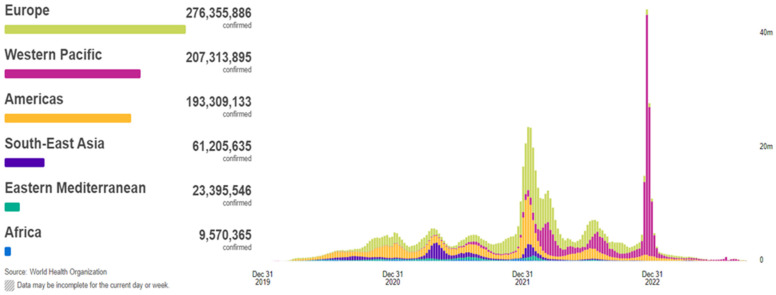
Situation by WHO Region, cases as of 26 September 2023 [2].

In 2019, the coronavirus disease, known as COVID-19, began to propagate, swiftly evolving into a global predicament of considerable magnitude. The COVID-19 outbreak precipitated an unforeseen and distressing scenario, ushering the entire world into the throes of a pandemic and resulting in the tragic loss of thousands of lives. According to the World Health Organization (WHO), COVID-19 has extended its reach to 220 countries and territories, with recorded infections and fatalities exceeding 771 million and around 7 million, respectively, as of 26 September 2023 (Figure 1). This devastating pandemic has had a profound impact on public health systems worldwide [1,2,3,4,5]. COVID-19, a novel pathogen emerging in 2019, had not been previously identified by humankind. Physical contact constitutes the primary mode of transmission for this disease among individuals. Infections are transmitted from the individuals afflicted with COVID-19 to healthy individuals through various means, including direct hand contact, mucous membrane contact, and respiratory exposure. The relentless global dissemination of COVID-19 has exacted a grievous toll, severely affecting critical areas such as public health, the global economy, and daily activities. Hence, the imperative of swift and precise detection of COVID-19 has become increasingly pronounced, given that infected individuals may go unnoticed and fail to receive timely and appropriate treatment [6].

The infection caused by severe acute respiratory syndrome coronavirus (SARS-CoV-2) leads to a wide range of clinical manifestations, from completely asymptomatic to severe acute respiratory distress syndrome, which complicates the diagnostic process [7,8]. The main barriers to containing the current COVID-19 outbreak are early detection and diagnosis. In this regard, it is extremely important to conduct a survey of the people affected by COVID-19 as soon as possible.

Generally, the diagnosing of COVID-19 can be achieved using three different methodologies: real-time reverse transcriptase-polymerase chain reaction (RT-PCR), chest CT imaging scan, and numerical laboratory tests [9].

RT-PCR tests are fairly quick, sensitive, and reliable. The sample is collected from a person’s throat or nose; some chemicals are added to remove any proteins, fats, and other molecules, leaving behind only the existing ribonucleic acid (RNA). The separated RNA is a mixture of a person’s RNA and the coronavirus RNA, if present. The RT-PCR test suffers from the risk of false negative and false positive results. Consequently, the spread of COVID-19 infection has increased because RT-PCR tests cannot immediately distinguish the infected people. Although several studies have observed that the sensitivity of chest CT in the diagnosing of COVID-19 is higher than that of RT-PCR, CTs and X-rays are not accurate tools for diagnosing COVID-19 [10,11]. There are some significant reasons: firstly, both chest CT and X-ray cannot accurately distinguish between COVID-19 and other respiratory infections. They can only point to signs of an infection, which could be due to other reasons, such as seasonal flu. Secondly, a huge number of patients infected with COVID-19 have normal chest CTs, which wrongly convince them that they are healthy. Moreover, the usage of the imaging equipment on COVID-19 patients is a critical hazard for doctors and other patients. CT scanners are complex and large machinery pieces [12]. They need to be carefully cleaned after each potential COVID-19 patient. However, even with precise cleaning, there is a high risk that the virus could remain on the surfaces in the CT scanner room. Because many countries and hospitals are not able to allocate sufficient testing resources, healthcare systems are deprived of one of their most effective tools for containing a pandemic: identification of case hotspots and targeted action toward regions and specific individuals with the disease. The scale of the testing shortage calls for methods for diagnosing COVID-19 that use the resources local healthcare facilities currently have. With increasing demand toward providing accurate tests, the dependency on CT images or RT-PCR tests as accurate tools for the detecting of COVID-19 patients is decreased dramatically. To this end, fast and accurate detection of COVID-19 patients is very important to prevent the spread of infection. Doctors may wrongly diagnose lung infection when most of the COVID-19 virus diseases are detected early enough.

Recently, machine learning is an adjunct tool for clinicians. Machine learning can automatically support medical diagnosis as a helping tool for identifying and detecting the novel coronavirus [13,14,15]. Hence, it makes perfect sense that the use of NLTs will provide more accurate diagnosis with a shorter waiting time. Machine learning techniques become more and more accurate over time, and they work on the same principle. Firstly, they receive some input training data. Then, they build the mathematical model depending on this training input data. Finally, the mathematical model is used to solve the problem at hand.

The Ghaderzadeh M. et al. review study presents a comprehensive assessment of the current status of all models utilized in the identification and diagnosis of COVID-19 using radiology techniques, particularly those relying on deep learning for processing [16]. The results suggest that deep-learning-based models possess exceptional capabilities to provide a precise and effective system for detecting and diagnosing COVID-19. Their application in the processing modalities would likely result in a substantial improvement in sensitivity and specificity values. Another article by Ghaderzadeh M. et al. highlights significant achievements in the application of artificial intelligence (AI) algorithms for detecting COVID-19. Notably, the AI algorithms used in radiology have demonstrated a remarkable sensitivity rate exceeding 95% and a specificity rate higher than 92% [17]. These AI algorithms outperform traditional radiological methods, offering a more accurate diagnostic tool. The Ghaderzadeh M. et al. study introduced a framework comprising two models based on convolutional neural networks (CNNs) leveraging transfer learning and parameter optimization [18]. The initial phase of this framework was tested and demonstrated outstanding results, achieving a detection sensitivity, specificity, and accuracy of 0.99, 0.986, and 0.988, respectively. In the second phase, the framework achieved even higher results with a sensitivity, specificity, and accuracy of 0.997, 0.9976, and 0.997, respectively.

In the article by Ali R.H. et al., a model for predicting the COVID-19 virus was introduced leveraging feature selection techniques [19]. This model encompassed three distinct stages: an initial preprocessing stage, followed by feature selection, and culminating in a classification stage. Their dataset comprised 8571 records, each with 40 distinct features, representing patients from 38 different countries. To discern the most influential features impacting the predictive capabilities of the model, they employed recursive feature elimination (RFE) and extra tree classifier (ETC) feature selection methods. Two distinct classification methods were subsequently applied to categorize these feature vectors, namely, the naive Bayes method and the restricted Boltzmann machine (RBM) method. The outcomes of their study were as follows: when classifying all features, naive Bayes achieved an accuracy of 56.18%, while RBM excelled, with an accuracy of 97.91%. For the top 10 features selected through the feature selection methods, naive Bayes improved to 66.32% and RBM exhibited exceptional accuracy at 99.92%. Notably, the RBM method consistently outperformed naive Bayes in terms of accuracy, establishing itself as the preferred choice for accurate classification.

In the article by Shanbehzadeh M. et al., the dataset comprises 501 case records categorized into two classes: COVID-19 and non-COVID-19. It encompasses 32 columns, each representing diagnostic features [20]. Various machine learning algorithms, including naive Bayes, Bayesian network, random forest (RF), multilayer perceptron, K-star, C4.5, and support vector machine, were used for analysis. The performance of these selected machine-learning models was subsequently assessed through the evaluation of several key performance metrics, including accuracy, sensitivity, specificity, precision, F-score, and receiver operating characteristic (ROC). The experimental findings unveiled that the RF algorithm exhibited exceptional diagnostic capabilities for COVID-19 screening, boasting an accuracy rate of 92.42%, specificity of 75.70%, sensitivity of 92.30%, precision of 92.40%, an F-score of 92.00%, and an impressive ROC score of 97.15%. Empirical evidence consistently demonstrated that the RF model outperformed the other six classification models in the study, establishing itself as the optimal choice for diagnostic purposes.

The study described by Huang C. et al. described a diagnostic strategy for COVID-19 known as feature correlated naive Bayes (FCNB) [21]. FCNB encompasses four distinct phases: the feature selection phase (FSP), feature clustering phase (FCP), principal feature weighting phase (MFWP), and the naive Bayes analysis with feature correlation (FCNBP) phase. In the initial stage, the FSP employs a genetic algorithm as a wrapper method to meticulously select the most effective features from laboratory test data, considering both COVID-19 and non-COVID-19 patients. Following this, the FCP generates numerous feature clusters based on the selected features derived from FSP, utilizing a clustering method. The pivotal FCNBP phase is dedicated to patient classification, deploying a weighted naive Bayes algorithm with multiple adjustments for feature correlations. This approach attains a remarkable detection accuracy rate of 99%.

In the work of Ahamad, Md.M. et al., a model was developed using supervised machine learning algorithms with the aim of identifying the key features that could serve as predictive markers for diagnosing COVID-19 [22]. The features under scrutiny encompassed a spectrum of information pertaining to the individuals in question, including age, gender, fever occurrence, travel history, and clinical data such as cough severity and instances of lung infections. In this comprehensive analysis, five distinct machine learning algorithms were applied: decision tree, random forest, gradient boosting machine, extreme gradient, and support vector machine boosting algorithms, all meticulously applied to the amassed dataset. The findings of this study revealed that the XGBoost algorithm consistently yielded the highest level of accuracy (>85%) when it came to both prediction and feature selection, effectively identifying the features that accurately indicated COVID-19 status across all age groups.

The study of Alotaibi A. et al. focused on the early prediction of disease severity using patient history and laboratory data, employing three distinct types of artificial neural networks (ANNs), support vector machines, and random forest regression with various learning methods [23]. Feature selection was executed through relief-based algorithms (RBAs), ultimately leading to the selection of 32 clinical features that were ranked based on their significance in predicting severity. Notably, 20 clinical features were excluded from consideration due to their adverse impact on the prediction process. Among the diverse methods applied, the random forest method, utilizing an ensemble of decision trees with “LP Boost”, “GentleBoost”, and “AdaBoostM1”, exhibited the highest overall performance. Subsequently, training a perceptron multilayer neural network with Bayesian regularization algorithms demonstrated the next best performance. Conversely, the RBF scores were the least effective, particularly in terms of sensitivity. The performance of GRNN and SVM, configured with various parameters, yielded satisfactory results.

As presented in article Chauhan H. et al., an artificial neural network served as the diagnostic model for assessing coronavirus positivity [24]. The researchers trained this model using a combination of clinically evaluated symptoms, patient-reported symptoms, case histories, and human exposure data. To enhance the performance of their classification model, the authors explored filter-based feature selection techniques, including chi-square, ANOVA F-score, and mutual information. Notably, the authors achieved the highest classification performance by training their model with features ranked according to the ANOVA F-score method. This yielded exceptional scores for accuracy, sensitivity, and F1 predictions, with values of 0.93, 0.99, and 0.94, respectively. Their study identified several key predictors of a COVID-19 diagnosis, with the most relevant ones encompassing the severity of sobbing, the severity of coughing, the presence of sobbing, the presence of coughing, fatigue, and the number of days since the onset of symptoms.

In the article by Srinivasulu A. et al., a function for predicting diseases caused by the COVID-19 virus was introduced [25]. This research incorporated convolutional neural networks and logistic regression, which are supervised learning strategies used to detect various diseases resulting from the COVID-19 virus. This system implemented an eight-pass detection approach to obtain accurate results. The study encompassed an examination of convolutional neural networks (CNNs) and logistic regression (LR) using datasets from the UCI database, Kaggle, and Google database. The authors devised a hybrid method specifically tailored for the COVID-19 virus and the analysis of various diseases. This involved dimensionality reduction using logistic regression (LR) followed by the utilization of a newly reduced function dataset for both convolutional neural networks and logistic regression. The proposed strategies exhibited impressive accuracy levels, reaching 78.82% for convolutional neural networks and an even higher 97.41% for logistic regression.

The aim of the study described by Ghaderzadeh M. et al. was to develop an efficient computer-aided detection (CAD) system for the COVID-19 virus using a neural search architecture network (NASNet) [26]. A local dataset included 10,153 computed tomography scans from 190 COVID-19-positive patients and 59 COVID-19-negative patients. Following training, hyperparameter optimization, and structural adjustments, the NASNet-based model achieved outstanding results in the test dataset, with a detection sensitivity, specificity, and accuracy of 0.999, 0.986, and 0.996, respectively. As a result, a CAD system was developed based on this model for COVID-19 detection using multiple lung computed tomography scans. It effectively distinguished all COVID-19 cases from non-COVID-19 cases without any errors. Overall, this deep-learning-based CAD system has the potential to significantly aid radiologists in early COVID-19 detection, accelerating disease identification and conserving healthcare resources during the pandemic.

In the study presented by Pourhomayoun M. et al., a dataset of over 2,670,000 laboratory-confirmed COVID-19 cases from 146 countries was used, including 307,382 labeled samples [27]. To address missing data, imputation methods were applied, encompassing mean/median/mode substitution and the K-nearest neighbors (KNN) technique. Subsequently, various filter and wrapper methods were utilized to select 57 out of 112 available features. Following the identification of the optimal feature subset, a range of machine learning algorithms, including support vector machine (SVM), neural networks, random forest, decision tree, logistic regression, and K-nearest neighbors (KNN), were employed to construct predictive models. The performance of each machine learning algorithm was assessed by comparing the accuracy metrics, ROC curves, and AUC values. Remarkably, the neural network algorithm outperformed the others, achieving an accuracy rate of 89.98% in this comprehensive evaluation.

New studies are constantly appearing in the literature, presenting various methods of diagnosing COVID-19 and distinguishing it from other diseases that cause changes in the lungs, such as pneumonia. These methods use different parameters from the study of patients. The most popular trend in the diagnosis of COVID-19 is the analysis of X-ray images [12,28,29,30] and CT scans [31,32,33,34]. These tests became the diagnostic basis at the beginning of the pandemic, which is confirmed by the research published in [35]. This research outlines a classification methodology employing radiomic features extracted from CT chest images to distinguish between the patients with coronavirus disease 2019 (COVID-19) and those with other forms of pneumonia. Proposed by Ibrahim D.M. et al., combining chest X-ray and CT images allowed them to obtain results of 98.05% accuracy (ACC), 98.05% recall, 98.43% precision, and 99.5% specificity using the VGG19 network [36]. However, it is important to note that these two approaches may not always be feasible for patient screening due to concerns related to radiation exposure, elevated costs, and the limited availability of the requisite medical devices.

Many methods have been provided for COVID-19 detection based on machine learning techniques. Despite the efficiency of these methods, they suffer from many limitations, such as low diagnosis accuracy, high complexity, and long prediction time. The work in this paper is concentrated on providing a new COVID-19 diagnosis system based on NLTs, which have proven to be the most effective methodology for COVID-19 diagnosis. The development of a disease prediction model based on clinical variables and standard clinical laboratory tests is proposed. The clinical variables include demographics, signs and symptoms, laboratory test results, and COVID-19 diagnosis. The variables include age, sex, serum levels of neutrophils, serum levels of leukocytes, serum levels of lymphocytes, reported symptoms (diarrhea, fever, coughing, sore throat, nausea, fatigue, etc.), body temperature, and underlying risk factors (renal diseases and diabetes).

The new contribution and value of the conducted study is that the authors have attempted to distinguish the cases of COVID-19 infection from the cases of pneumonia. This is a far more difficult task than distinguishing COVID-19 virus infection from completely healthy cases. The latter task is often described in the literature, while from the point of view of complexity, it is much easier than the one presented. Furthermore, the proposed method of diagnosis is based on a comprehensive set of diagnostic information using multiple sources of information. This approach is more reliable compared with the proposed solutions based on a single source of information such as radiological imaging.

## 2. Materials and Methods

### 2.1. Research Material

The COVID-19 dataset is a real dataset that is used to detect COVID-19 patients. These raw hospital data contain the results of clinical laboratory tests and clinical variables collected from different cases who were admitted to the hospital (Vinnitsa, Ukraine). The clinical variables include demographics, symptoms, laboratory test results, and COVID-19 diagnosis. The variables include blood test results (various blood properties), reported symptoms (diarrhea, fever, coughing, sore throat, nausea, fatigue, etc.), body temperature, anamnesis data, and underlying risk factors (renal diseases and diabetes). The total number of cases in this real dataset is 128. According to this dataset, it is considered two class categories, called COVID cases and non-COVID cases, as shown in Table 1.

**Figure 2:**
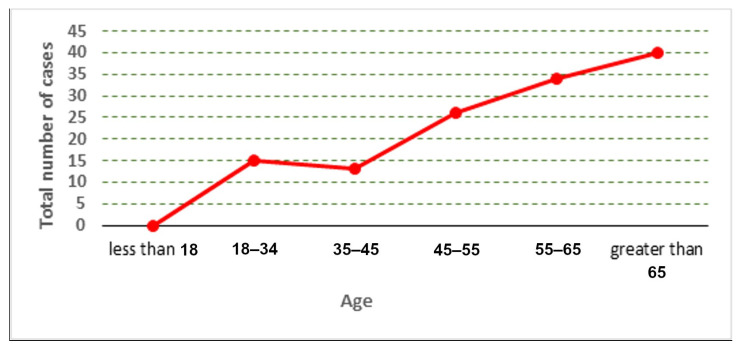
Total number of cases according to age.

**Figure 3:**
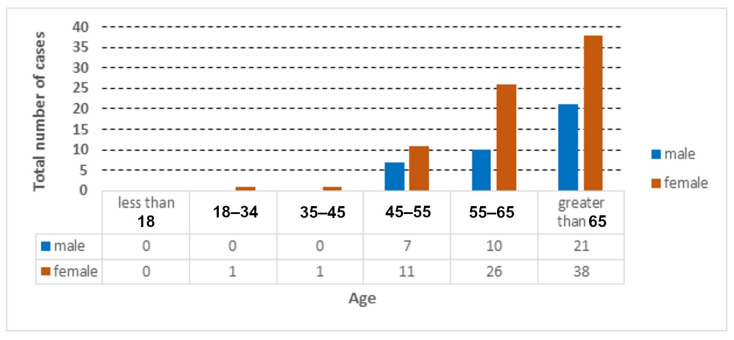
Total number of COVID-19 cases according to age and gender.

**Figure 4:**
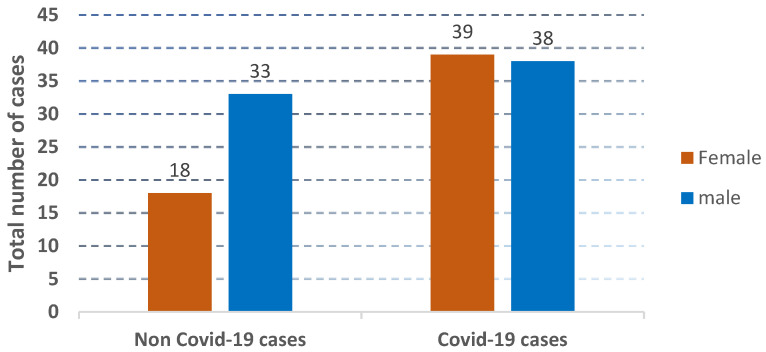
The presentation of the distribution of non-COVID-19 cases and COVID-19 cases.

The distribution of the cases used in the collected dataset is represented according to age and gender, as shown in Figure 2, Figure 3 and Figure 4.

The important features of basic information, symptoms (fever, cough, muscle soreness), diagnostic results, prior disease, symptom history (pneumonia, diarrhea, runny nose) that are directly or indirectly related to the COVID-19 disease were extracted. The patients did not all develop the same symptoms; symptoms of every patient were found individually. In detail, some keywords for each feature were selected, and then those keywords were matched with text data and the features were extracted individually. However, much of the data was in the form of unstructured text information, which can be difficult to process. The data contained patient symptoms in a text format. Categorical data were converted to dummy variables, because non-numerical data are not allowed in the adopted machine learning algorithm. A data transforming process was applied through transforming the data into numeric forms (“0” and “1”). The true value (“1”) means the existence of the feature on a patient, while the false value (“0”) means the absence of the feature. Lastly, a final dataset, which contained the main features, was generated. In this dataset, most of the values were binary, and several fields were numerical values. Brief descriptions of some features that affect COVID-19 patients are shown in Table 2.

### 2.2. Data Preprocessing

**Figure 5:**
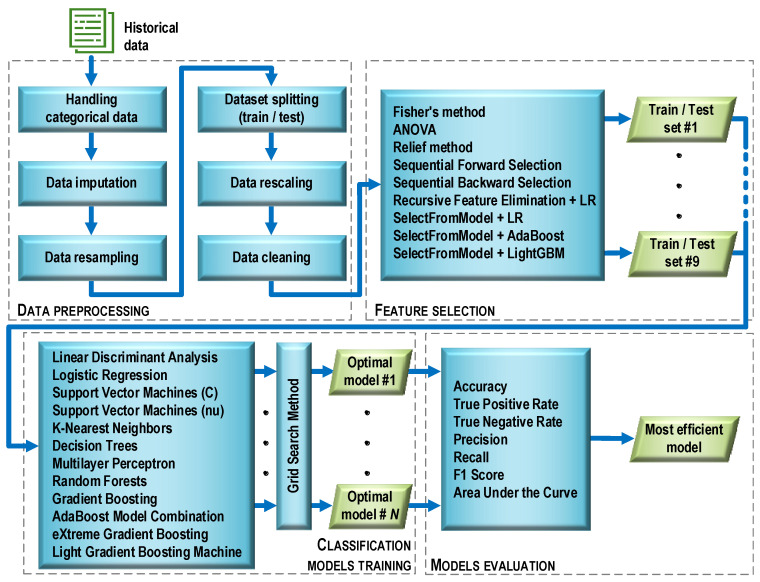
Machine learning process flow.

Before the essential steps of machine learning began, the data were preprocessed (Figure 5). This stage consisted of six operations characterized below.

**Handling categorical data.** The original data contained a number of categorical features with text labels. In contrast, most machine learning algorithms require numeric feature values to work properly. Therefore, the categorical data were encoded with dummy variables. After this operation, the value of each categorical feature was “1” if there was a symptom associated with the feature, or “0” otherwise.

**Data imputation.** The initial dataset contained missing values (about 10%). Such a situation can cause errors during data analysis. To avoid this, various methods of data imputation are used. One of the most popular methods, referred to as replace with the summary, was applied. Missing categorical values were replaced with the value occurring most often (mode), while continuous values were replaced with the average value.

**Data resampling.** Initially, the dataset consisted of 128 cases. Its structure was such that there were 77 cases (60%) diagnosed with the COVID-19 disease and 51 cases (40%) with diagnosed pneumonia (non-COVID-19). The dataset was unbalanced, which could risk biasing the prediction of the majority class by classifiers built on its basis. To avoid this danger and ensure a balanced number of positive and negative observations, a resampling method known as a synthetic minority oversampling technique (SMOTE) was used. The SMOTE technique is based on the fact that the size of the minority class is increased by generating new synthetic cases by combining all K-nearest neighbors (by default, K = 5) of the minority class using similarities in feature space (Euclidean distance). After applying the SMOTE technique, the dataset counted 154 cases, with both classes (COVID-19 and non-COVID-19) having 77 cases each.

**Dataset splitting (train/test).** The full dataset (154 observations) was randomly divided into a training and a testing part in such a way that the training part accounted for 70% and the testing part for 30% of the full set. During the division, the same proportion (50%) between observations belonging to both classes was maintained as in the full set. Details of the size of each subset are provided in Table 3.

**Data rescaling.** Ninety-nine feature descriptors were obtained as a result of assessing the patients’ diagnosis parameters. Due to the fact that the feature descriptors were measured on different scales (quotient, nominal), the features were rescaled using classical standardization: $z_{ij}=(x_{ij}-{\bar{x}}_{j})/s_{j}$, where: *x_ij_* is the value of feature *j* for observation *i*; *z_ij_* is the standardized value of feature *j* for observation *i*; ${\bar{x}}_{j}$ is the arithmetic mean of feature *j*; and *s_j_* is the standard deviation of feature *j*. After standardization, the interval scale and normal distribution *N*(0,1) were in effect for all features. Scaling was performed once for the training data. Then, the test data were transformed in a similar manner. During this process, the mean and variance vectors obtained during the standardization of the training data were used for each feature.

**Data cleaning.** A four-step data cleaning procedure was carried out:

- Removal of the features taking constant values (variance equal to 0);
- Removal of the features assuming almost constant values (variance less than 0.01);
- Removal of the features assuming duplicate values;
- Removal of the mutually correlated features. The Pearson correlation coefficient (*r*) was used, which detects linear dependencies between features and assumes normality of their distribution. The features for which |*r*|> 0.33, indicating the presence of a moderate and strong correlation, were removed.

**Figure 6:**
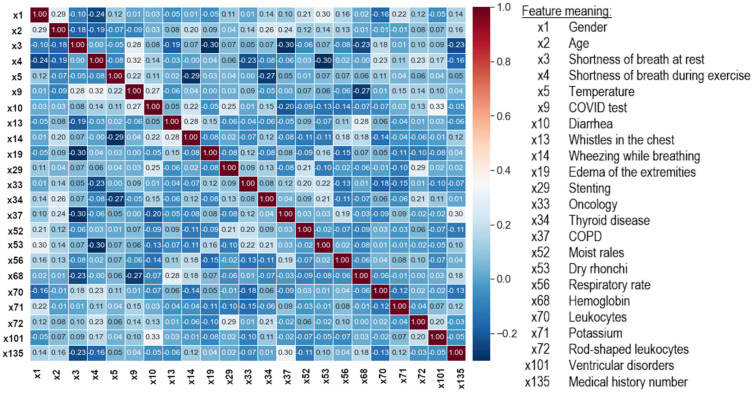
Correlation matrix between the output features of the dataset cleaning process.

As a result of the data cleaning, the number of features was reduced from 99 to 23 (Figure 6). Data preprocessing was performed using the scikit-learn library and the Python programming language, which was used in further experiments.

### 2.3. Feature Selection

In the general case, the purpose of feature selection is to restrict the original (complete) set of features to a certain subset that includes the features that are important for the criterion used. As a result of data cleaning, the full set of 99 features was reduced to 23 features. Such a significant reduction was mainly due to the presence of a large number of features with moderate and strong cross-correlation. Therefore, at the selection stage, an additional reduction was abandoned, and the goal was to build a ranking list for each of the selection methods used. The ranking lists contained a set of features in the order that reflects their importance in the process of identifying the observations related to one or another diagnosis. As a result of this processing step, nine training sets and nine test sets were obtained.

Nine feature selection methods were used. Their acronyms are given in brackets.

Filter methods:
  - Univariate Fisher’s method (FISHER) and the method of analysis of variance (ANOVA);
  - Multivariate Relief method (RELIEF).
Wrapper methods:
  - Sequential forward selection (SFS);
  - Sequential backward selection (SBS);
  - Recursive feature elimination along with LogisticRegression estimator (RFE).
Embedded methods:
  - SelectFromModel method with logistic regression (LR) evaluation;
  - SelectFromModel method with AdaBoost (ADA) evaluation;
  - SelectFromModel method with LightGBM (LGBM) evaluation.

### 2.4. Classification Models Training

The goal set in the research was to solve a binary classification task, which belongs to supervised learning problems. Let us give a general formulation of the problem of learning with a teacher. Let *X* be the general set of objects and *Y* be the set of possible labels of objects connected by some unknown dependency *y*: *X* → *Y*. The task of supervised learning is to construct an algorithm *C* that approximates the *y* dependence on a known training sample *T* = {(*x*_1_, *y*_1_), …, (*x_m_*, *y_m_*)} according to some measure of quality *Q*. In the case of a classification problem, each object is described by some discrete variable that the algorithm tries to predict; that is, to predict the class of the object. The simplest classification task is binary classification, in which there are two classes of objects.

During the study, the grid search method was used in the training process, which made it possible to determine the optimal values of the hyperparameters of each model. The GridSearchCV method, available in the model_selection module of the scikit-learn library, was used for this purpose. The models were evaluated based on 10-fold cross-validation. Each model, which was considered optimal in terms of a given number of features, was saved as a file on a disk. Then, the best model was selected from the available set (i.e., the one that provided the highest classification accuracy with the minimum number of features). Such a model is named as optimal in Figure 5. The selection of the best model also made it possible to determine the optimal set of features that was used in the training process. The procedure above was used for all filter and wrapper methods. For embedded methods, only those models that were used in the feature selection process were trained and tuned.

Twelve different supervised learning methods were used to build the classifiers. Their acronyms are given in brackets.

Methods to build a single model:
  - Linear discriminant analysis (LDA);
  - Logistic regression (LR);
  - Support vector machines with the C regularization parameter (SVM);
  - Support vector machines with the nu parameter to control the number of support vectors (NuSVM);
  - K-nearest neighbors (KNN);
  - Decision trees (DT);
  - Multilayer perceptron (MLP).
Ensemble methods:
  - Random forest (RF);
  - Gradient boosting (GRADBoost);
  - AdaBoost model combination (ADABoost);
  - eXtreme gradient boosting (XGBoost);
  - Light gradient boosting machine (LGBM).

### 2.5. Model Evaluation

**Figure 7:**
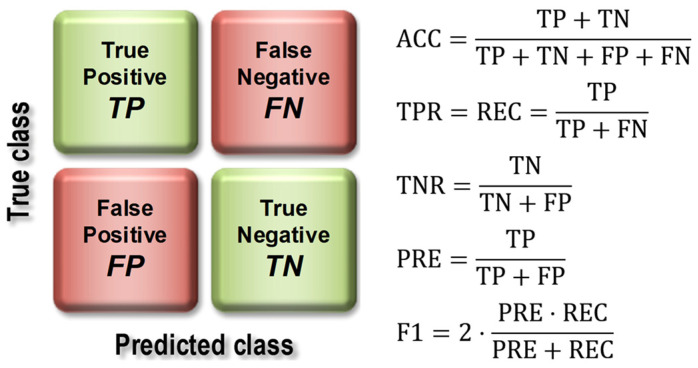
Construction of the confusion matrix and how to use it to calculate model quality indices.

The basis for calculating the value of model quality metrics was the confusion matrix (Figure 7), the elements of which represent the number of false positive (FP), false negative (FN), true positive (TP), and true negative (TN) cases.

**Figure 8:**
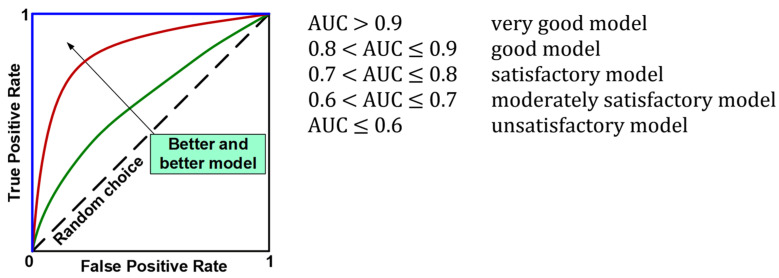
ROC curves and interpretation of AUC values.

The evaluation of the models using the test set resulted in the calculation of overall classification accuracy (ACC), sensitivity (TPR, Recall, REC), specificity (TNR), precision (PREC), F1 index (F1), and area under the ROC curve (AUC). Figure 7 shows the formulas used to calculate the above metrics, while Figure 8 provides an interpretation of the ranges of AUC index values. During the evaluation of the models, ROC curves were also plotted (Figure 8) and confusion matrices were constructed showing detailed information on the recognition of each class. For more information on calculating classification quality performance metrics, see [37,38,39], among others.

## 3. Results

The number of features in the lists built by the separate methods was as follows: filter and wrapper methods—23; embedded methods: logistic regression—8, AdaBoost—6, LGBM—23 (Table 4). The selection of the optimal number of features for each of the selection methods used was carried out at the next stage, classification models training. Feature selection was performed only on the training data; then, its results were used to transform the test data.

As Table 4 shows, different feature selection methods selected a different number of features and various features as the most relevant to detect COVID-19. Fisher is one of the filter methods that was applied for selecting the 23 features. The second column of the table presents the sorting of 23 important features using Fisher. It is viewed as ranking; the first feature is the most important feature, while the remaining positions mean the rank of each feature depending on its importance. The other feature selection methods demonstrate the importance of features selected by them. It should be noted that age (X2), gender (X1), shortness of breath at rest (X3), respiratory rate (X56), and COPD (X37) features are in the list of five most relevant features according to the Fisher method. The ANOVA method selected the following features: age (X2), gender (X1), shortness of breath at rest (X3), oncology (X33), and leukocytes (X70) as the five most important features. Respectively, the five most important features selected by each feature selection method can be seen in the table. It should be emphasized that features such as age (X2), gender (X1), shortness of breath at rest (X3), respiratory rate (X56), hemoglobin (X68), and leukocytes (X70) are found in almost all methods among the most important features.

In the process of training the classification models, nine training sets (one for each selection method) were used, arranged in accordance with the ranking of features. It should be emphasized that in this scenario of the study, the full set of features was used; i.e., the one that was returned by a given selection method. While the models were being trained, the number of features in the training set changed according to the ranking list. For the filter and wrapper methods, this number varied from 2 to 23; for logistic regression, from 2 to 8; for the AdaBoost method, from 2 to 6; and for the LGBM method, from 2 to 23.

**Figure 9:**
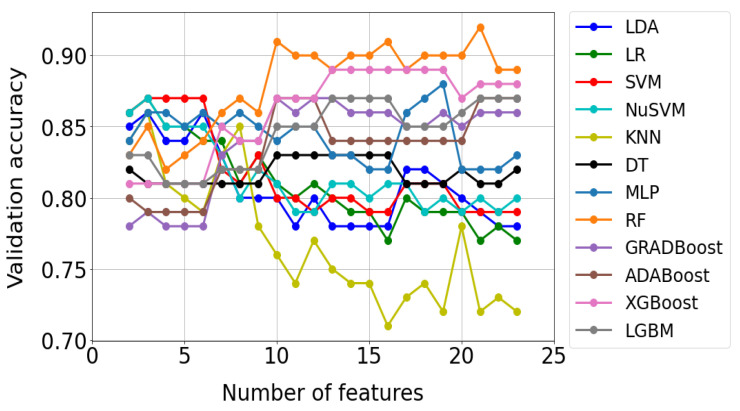
Validation results of optimal models for the FISHER selection method. Optimal models were selected using the grid search method.

Figure 9 shows an example of test results for the Fisher method. Each of the 12 curves in the graph corresponds to a different classification method. In turn, the points marked on the curves reflect the accuracy of the model check, which turned out to be optimal for a given number of features. As can be seen from Figure 9, 22 optimal models were built for each classification method. A total of 264 such models were built using all methods and saved as files to disk.

On the basis of the results presented in Figure 9, the most efficient model was selected for each classification method; i.e., the one that provided the highest validation accuracy with the minimum number of features in the training set. The selection of the most efficient model clearly defined the optimal set of features that was used in its construction. In Table 5, the results of selecting the most efficient models for each classification method are presented. On the basis of data analysis, the RF model (the row highlighted in gray in Table 5) was found to be the best for the Fisher method.

The criterion for selecting the best model was the same as for selecting the optimal model; i.e., the highest validation accuracy with the minimum number of features in the training set. For the RF model, the validation accuracy was 0.92, and 21 features were used. For all filter and wrapper methods, a model selection procedure was used that was previously characterized using the Fisher method. In the case of embedded methods, only those classifiers that were used in the selection process were constructed and configured.

The summary results are presented in Table 6. For each model, the selection method used to rank the features, the classification method, and the number of features used are indicated. The symbols appearing in the model designation should be interpreted as follows: first position—selection method (A-ANOVA, F-FISHER, R-RELIEF); second position—classification method; third position—the number of features used.

**Figure 10:**
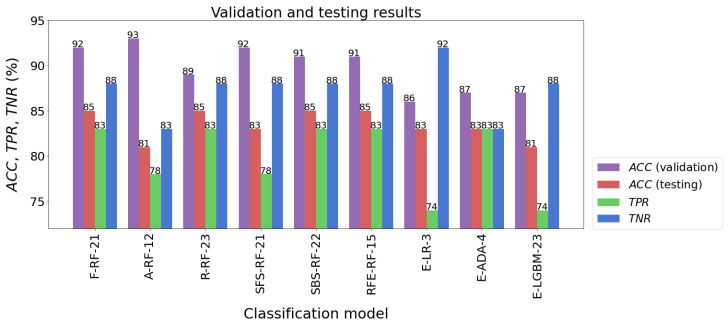
Validation and testing results of the best classification models.

When several learning algorithms were used, many different models were evaluated. From these, the most effective model was selected, which could then be used to forecast future new data. It should be added here that the parameters of the procedures used for data preprocessing, such as feature scaling or dimensional reduction, were determined solely on the basis of the training set. Then, they were used to transform test data and new data. The following models had the highest classification accuracy for the test sample: F-RF-21 (ACC = 85%), R-RF-23 (ACC = 85%), SBS-RF-22 (ACC = 85%), and RFE-RF-15 (ACC = 85%). The accuracy of testing other models was not much worse, and fluctuated between 81% and 83%. The results of the models’ validation are listed in Table 7 and presented in Figure 10.

**Figure 11:**
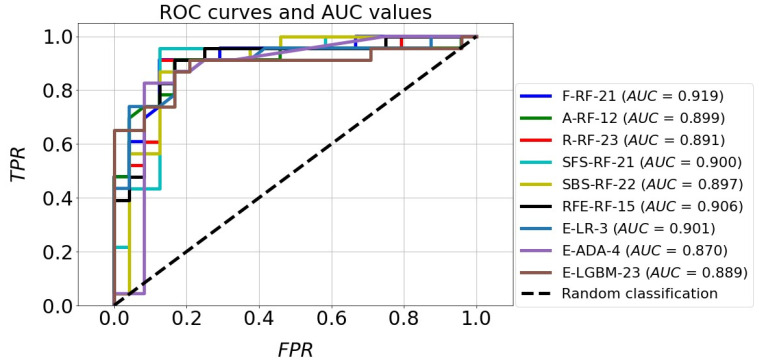
ROC curves and AUC values of the best classification models.

The classification accuracy of all the models for the test set exceeded the level of 80%. It should also be noted that almost all models achieved a higher TNR than TPR. The high quality of the four models mentioned above is also confirmed by the ROC curves in Figure 11. In the plot (FPR, TPR), they pass much closer to the point with coordinates (0,1), which is an ideal classification, compared with the corresponding curves of the other models. This fact is confirmed by the size of the areas under the ROC curves expressed by the AUC parameters. For the top four models mentioned above, the AUC value ranges from 0.891 to 0.919, while for the rest of the models the range is 0.870 to 0.901. Although the accuracy scores of these four identified effective models are the same, attention needs to be paid to how many parameters they implement. The RFE-RF-15 model works with 15 features, so it is the most efficient one. Also, the E-ADA-4 model can be considered as optimal, because compared with the RFE-RF-15 model, its accuracy was 2% weaker (ACC = 83%). Its other classification quality metrics were somewhat lower than those of the RFE-RF-15 model, and the number of features was minimal and equal to four.

**Figure 12:**
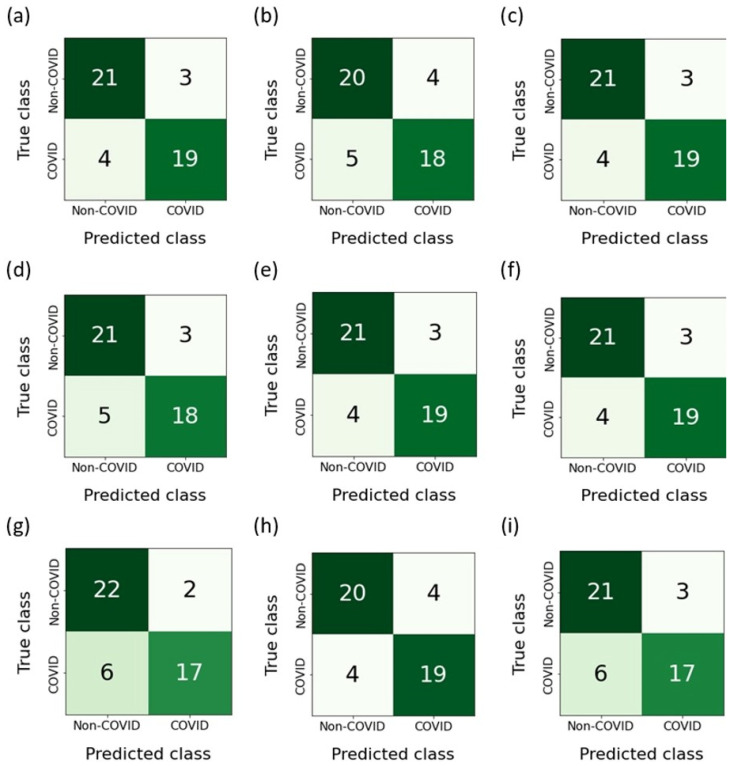
Confusion matrices: (**a**) F-RF-21; (**b**) A-RF-12; (**c**) R-RF-23; (**d**) SFS-RF-21; (**e**) SBS-RF-22; (**f**) RFE-RF-15; (**g**) E-LR-3; (**h**) E-ADA-4; (**i**) E-LGBM-23.

Figure 12 shows the confusion matrices built as a result of the classification of features belonging to the test sample. The RFE-RF-15 model, which proved to be the most effective, misdiagnosed ordinary pneumonia (non-COVID-19) as COVID-19 three times. On the other hand, when diagnosing COVID-19, the number of errors was four. Taking into account the E-ADA-4 model, it was mistaken four times when diagnosing COVID-19 and four times when diagnosing pneumonia.

## 4. Discussion

The RFE-RF-15 model was created using 15 features. In Table 8, these features are sorted according to their importance. It is clear from the table that age and shortness of breath at rest are the highly relevant features to a positive diagnosis of COVID-19. The mean age of individuals is 63.9 years among COVID-19 patients and 44.3 years in non-COVID-19 cases. The shortness of breath at rest was the second most important symptom, with 92.2% patients with COVID-19. According to RFE-RF-15, the top 10 relevant features are age, shortness of breath at rest, leukocytes, granulocytes, COVID test, COPD, shortness of breath during exercise, potassium (K), diabetes mellitus, and diarrhea. Table 8 shows that 12 patients (15.6%) had COPD, 61 patients (79.2%) had a shortness of breath during exercise feature, and 12 patients (15.6%) had diabetes mellitus in the COVID-19 positive cases. Table 9 demonstrates four important features selected by the E-ADA-4 model: N IB, age, respiratory rate, and hemoglobin. Considering both tables (Table 8 and Table 9), it is also seen that age, hemoglobin, and N IB features occur in both methods; this confirms the importance of these features in predicting COVID-19.

Among the advantages of the conducted study is that, unlike many studies presented in the literature, the obtained results are not on distinguishing the cases of COVID-19 disease infection from healthy cases, which seems like an easy task, but on distinguishing the cases of COVID-19 infection from cases of pneumonia. This task is definitely more difficult and remains a real challenge in the practice of COVID-19 disease diagnosis. The information obtained through medical history, demographic data, signs and symptoms of the disease, as well as laboratory test results was used. These data form a comprehensive set of diagnostic information that is far more reliable than any other data, such as those based only on radiological imaging. In contrast, some drawbacks of the conducted study include the relatively small number of observations used (128) and the unbalanced original dataset, which necessitated the use of the resampling technique.

Comparing the obtained results with studies published in the literature, significant differences related to the material used in the research can be seen. One of the best results presented in the literature is the combination of chest x-ray and CT images proposed in [36]. The utilization of convolutional networks, widely acclaimed in the realm of medical imaging diagnostics, has yielded remarkable outcomes, with an accuracy rate (ACC) of 98.05%, recall rate of 98.05%, precision rate of 98.43%, and specificity rate of 99.5%, all achieved through the deployment of the VGG19 network. Notably, it is imperative to highlight that these findings from the multi-classification deep learning model enable the discrimination of COVID-19 from both pneumonia and lung cancer.

Studies published present a classification method that leverages radiomics features extracted from CT chest images to distinguish the patients with COVID-19 from those with other forms of pneumonia [35]. For the COVID-19 patients, correlation analyses were conducted to determine whether these textural and histogram features correlated with key laboratory test indices, including blood oxygen levels, white blood cell counts, lymphocyte counts, neutrophil counts, and C-reactive protein levels. The final model exhibited strong discriminatory capabilities when validated independently, achieving an accuracy of 89.83%, sensitivity of 94.22%, specificity of 85.44%, and an impressive area under the curve (AUC) of 0.940. Nevertheless, it is important to note that while image analysis is a powerful tool, it may not always be suitable for patient screening due to the concerns related to radiation exposure, high costs, and limited device availability. Another approach discussed in the literature involves the utilization of ECG signals [40]. ECG data labeled as either COVID-19 or normal were classified to assess the model’s ability to detect COVID-19. According to the results, this proposed approach demonstrates promising performance in COVID-19 detection, achieving an accuracy of 96.20% and an F1 score of 96.30%.

The study by Zhao B. et al. tested the usefulness of vibrational spectroscopy combined with machine learning for early screening of COVID-19 patients [41]. The authors introduced a novel hybrid classification model known as the grey wolf optimized support vector machine (GWO-SVM). For the unknown vibrational spectra, the GWO-SVM model demonstrated remarkable results, achieving an accuracy of 0.9825, specificity of 0.9714, and an F1 score of 0.9778 for the saliva FTIR spectra dataset.

Another avenue in COVID-19 diagnostics involves laboratory tests, which have garnered substantial validation in numerous studies for their significant diagnostic efficacy. Routine blood tests present a cost-effective and rapid means of COVID-19 detection. These precise algorithms can prove highly effective, especially during the peak of a pandemic when hospitals are inundated with patients. The research presented by Chadag K. et al. shows that the use of machine learning methods allows achieving satisfactory diagnostic results [13]. In this research, RF, LR, KNN, and XGBoost supervised classifiers were utilized for the prediction of the deadly virus. The best results were obtained for the random forest model: accuracy of 92%, sensitivity of 71%, and specificity of 96%. The aim of the Huyut M.T. et al. research was to evaluate the diagnosis and prognosis of COVID-19 based on blood gas data by applying a chi-square automatic interaction detector (CHAID) decision tree model [42]. The decision tree, constructed with five key variables, demonstrated an overall classification accuracy of 68.2%. This translated to correctly identifying 59.5% of patients with COVID-19 and 77.0% of individuals without COVID-19. In a study [43] utilizing the LogNNet model, 12 crucial biomarkers for prognosticating COVID-19 patients with varying degrees of infection severity were identified. The LogNNet model exhibited an overall accuracy of 90.9%. When it came to diagnosing mildly infected patients, the accuracy rate soared to an impressive 99.0%. However, for severe cases, the accuracy rate dropped to 36.6%. Nonetheless, the effectiveness of the LogNNet model in correctly distinguishing between mild and severe patients, as indicated by their actual conditions (recall value), remained substantial. It achieved recall values of 91.4% and 83.1% for mild and severe patients, respectively. Other studies based on the use of laboratory data and machine learning achieved results at the level of 75–86% for accuracy [44,45,46,47,48].

A novel diagnostic model for COVID-19 has been developed that significantly improves the overall prediction accuracy [49]. The datasets encompass a range of critical information, including demographic data such as age, gender, and comorbidities, alongside essential diagnostic details and related tests. These encompass symptoms, vital signs, laboratory results, values obtained from routine blood tests, and chest CT imaging findings as well as information regarding patient disposition, ICU admissions, and hospital discharge outcomes. The proposed methodology consists of two primary stages. In the initial phase, the relative total feature contribution importance estimation factor (RTF-C-IEF) is employed to assess the significance of individual features. Subsequently, the modified flamingo search algorithm is utilized in the second stage to select a set of relevant and non-redundant features. Finally, a support vector machine (SVM)-based classifier is applied to predict COVID-19 using the carefully chosen clinical text features. This allows achieving classification accuracy at the level of 95.05%.

When analyzing the studies presented in the literature, it can be seen that the use of clinical and laboratory data has great diagnostic potential. The number of publications proves the intensive search for the algorithms that give better and better results and the importance of this diagnostic method. The results presented in this article do not differ in effectiveness from others presented in the literature. This proves the potential of the method used. This method can become an alternative to expensive imaging tests (Table 10).

**Figure 13:**
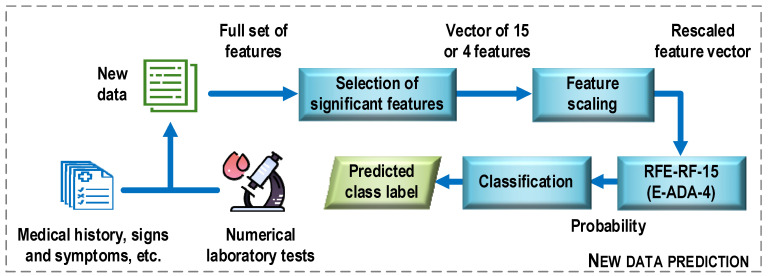
General structure of the COVID-19 case detection system.

Figure 13 shows a general structure of the COVID-19 case detection system. First, new data is preprocessed. Examples of operations that can be performed at this stage include converting descriptor text options to numeric ones, recovering missing data, and removing multicollinear features. Next, the procedure for selecting the most important features for the construction of the optimal classifier models is performed. It means here only the set of features that was used to train the most efficient classification model. In the next step, the selected features are scaled (e.g., standardized) using the same mean and variance vectors that were used to scale the training sample. The last step is to classify the new data. Here, a classification model is used, which turns out to be the most effective at the evaluation stage in relation to the test sample. In the considered case, the results of the experiments allow proposing two classifier models, the RFE-RF-15 and E-ADA-4 models. On the basis on these models and the values of the feature descriptors, class labels are assigned to the analyzed data (prediction).

## 5. Conclusions

The main result of the study corresponds to the feature vectors obtained on the basis of numerical clinical laboratory tests, which, together with the proposed classification methods, can be an element of a computer system that supports the doctor in the diagnostic process. This system will allow the classification of analyzed cases into patients who need to detect cases of COVID-19. Nine of the constructed models achieved classification accuracy at a level exceeding 80%. On the basis of the results obtained, two models were adopted as a proposal for the final solution of the problem of automatic data classification, supporting the doctor in the diagnosis of the infectious disease COVID-19. They are RFE-RF-15 and E-ADA-4, since these models had higher accuracy than other models and worked with fewer features. The first is a classifier built using the random forest. This model uses 15 features selected using the recursive feature elimination along with logistic regression estimator (RFE) method. For the data belonging to the test set, this resulted in accuracy of 0.85 (85%), sensitivity of 0.83, and specificity of 0.88. The second model was built using the SelectFromModel method with AdaBoost classifier, which resulted in accuracy of 0.83 (83%), sensitivity of 0.83, and specificity of 0.83. This model uses four features.

The study used three filter methods (univariate-Fisher’s method (FISHER), the method of analysis of variance (ANOVA), and multivariate-relief method (RELIEF)), three wrapper methods (sequential forward selection (SFS), sequential backward selection (SBS), and recursive feature elimination along with LogisticRegression estimator (RFE)), and three embedded methods (SelectFromModel method with logistic regression (LR) evaluation, SelectFromModel method with AdaBoost (ADA) evaluation, and SelectFromModel method with LightGBM (LGBM) evaluation). Each method of feature selection selected a different number of features. Twenty-three features were selected by the filter and wrapper methods; with the embedded methods the results were as follows: logistic regression—8 features, AdaBoost—6 features, and LGBM—23 features. The features revealed by the filter and wrapper methods were used by 12 supervised machine learning methods. These were: linear discriminant analysis (LDA), logistic regression (LR), support vector machines (SVM), support vector machines with the *nu* parameter to control the number of support vectors (NuSVM), *K*-nearest neighbors (KNN), decision trees (DT), and multilayer perceptron (MLP). In addition, five ensemble methods were used: random forests (RF), gradient boosting (GRADBoost), AdaBoost model combination (ADABoost), eXtreme gradient boosting (XGBoost), and light gradient boosting machine (LGBM).

The plan for further work in the scope presented in the dissertation includes attempts to create a complete system for diagnosing infectious COVID-19 diseases based on clinical and laboratory data. First of all, the developed algorithm needs to be tested on a larger number of patients. This is one of the most problematic issues when looking for new solutions in medical diagnostics. Difficulties in accessing relevant data-protected patient databases and finding results that meet strict criteria usually force investigators to conduct studies on fewer samples. When building systems that aim to create solutions commonly used in healthcare settings, extensive testing must be performed to ensure that the system works correctly and fulfills its diagnostic roles and procedures. Another important aspect of the process of creating a tool for accurately diagnosing COVID-19 infectious diseases is the inclusion in the predictive system of a greater number of classes to which the test samples are assigned.

The authors intend to continue the research presented in this paper. Within this framework, collecting a larger and more balanced dataset is planned. In addition, the CSDI collection will be expanded to include textural features of patients’ radiological images, which are likely to improve the predictive accuracy of the diagnostic models. To support data collection in a database that integrates diverse diagnostic information, a dedicated computer application will be built. The obtained results can be used to solve the problems of selecting informative features and classifying laboratory and clinical data in medicine and biology.

## Figures and Tables

**Table 1:** Demographic characteristics of patients.

Characteristics	Value
Non-COVID-19 cases (n = 51)	
Age (years), mean (SD)	44.3 (12.8)
Female sex, %	35.3%
Male sex, %	64.7%
COVID cases (n = 77)	
Age (years), mean (SD)	63.9 (10.1)
Female sex, %	50.6%
Male sex, %	49.4%
Total number of cases (n = 128)	
Age (years), mean (SD)	56.1 (14.8)
Female sex, %	44.5%
Male sex, %	55.5%

**Table 2:** Brief descriptions about the features.

No	Feature Name	Type	Description
1	Age	Continuous	To determine the age of the person
2	Gender	Categorical	To determine male or female
3	Diabetes	Categorical	Whether patient has diabetes
4	COPD	Categorical	Chronic obstructive pulmonary disease
5	Smoker	Categorical	Whether patient is a smoker
6	Wheezes	Categorical	Sound made while breathing
7	Cough	Categorical	Whether patient has cough symptoms
8	Fever	Categorical	Whether patient has fever
9	Diarrhea	Categorical	Whether patient has diarrhea
10	Fatigue	Categorical	Whether patient is experiencing fatigue
11	Headache	Categorical	Whether patient is experiencing a headache
12	Temperature	Continuous	Body temperature, °C
13	Pulse rate	Continuous	Number of heart beats per minute (bpm)
14	Sys	Continuous	Systolic blood pressure measured in mmHg
15	Dia	Continuous	Diastolic blood pressure measured in mmHg
16	Respiratory rate	Continuous	Respiratory rate measured in breaths per minute
17	Sats	Continuous	Oxygen saturation
18	COVID test results	Categorical	COVID-19 test results
19	Hemoglobin	Continuous	Level of hemoglobin in blood
20	Lymphocytes	Continuous	Lymphocytes per microliter of blood

**Table 3:** The result of splitting the full dataset into training and testing parts.

Dataset	No. of Observations	Part of the Full Dataset	COVID-19	Non-COVID-19
Full	154	1.0	77	77
Training	107	0.7	54	53
Testing	47	0.3	23	24

**Table 4:** Ranking lists returned by each feature selection method.

No.	Filter Methods			Wrapper Methods			Embedded Methods		
	FISHER	ANOVA	RELIEF	SFS	SBS	RFE	LR	ADA	LGBM
1	X2	X2	X13	X2	X2	X2	X2	X135	X1
2	X1	X1	X53	X68	X68	X3	X3	X2	X2
3	X3	X3	X3	X1	X14	X70	X70	X56	X3
4	X56	X33	X52	X3	X34	X72	X9	X68	X4
5	X37	X70	X5	X33	X1	X9	X4	X70	X5
6	X29	X34	X9	X13	X70	X37	X72	X72	X9
7	X135	X37	X14	X5	X56	X4	X71		X10
8	X53	X29	X56	X29	X101	X71	X37		X13
9	X71	X101	X70	X10	X4	X34			X14
10	X68	X135	X33	X4	X10	X10			X19
11	X13	X9	X10	X14	X29	X53			X29
12	X4	X68	X34	X9	X19	X13			X33
13	X9	X53	X37	X19	X52	X68			X34
14	X10	X5	X101	X70	X13	X29			X37
15	X101	X71	X71	X34	X37	X135			X52
16	X34	X13	X68	X71	X53	X14			X53
17	X72	X4	X72	X53	X72	X19			X56
18	X33	X19	X29	X37	X9	X101			X68
19	X5	X72	X4	X56	X33	X5			X70
20	X52	X14	X135	X135	X3	X33			X71
21	X70	X56	X19	X72	X135	X52			X72
22	X14	X52	X2	X52	X5	X1			X101
23	X19	X10	X1	X101	X71	X56			X135

**Table 5:** Results of model selection that were found to be most effective for each classification method using the FISHER selection method.

Classification Method	Validation Accuracy	Optimal Number of Features	Optimal Model Parameters
LDA	0.86	3	Solver = ‘svd’
LR	0.87	3	C = 100.0, penalty = ‘l2’, solver = ‘lbfgs’, tol = 1.0
SVM	0.87	3	C = 1.0, gamma = ‘scale’, kernel = ‘rbf’
NuSVM	0.87	3	Gamma = ‘scale’, kernel = ‘rbf’, nu = 0.4
KNN	0.86	3	n_neighbors = 3
DT	0.83	10	Criterion = ‘gini’, max_depth = 5
MLP	0.88	10	Activation = ‘relu’, alpha = 0.01, solver = ‘lbfgs’, hidden_layer_sizes = (7,), max_iter = 2000
RF	0.92	21	max_depth = 10, n_estimators = 50
GRADBoost	0.87	10	loss = ‘deviance’, n_estimators = 50
ADABoost	0.87	10	n_estimators = 10
XGBoost	0.89	13	eval_metric = ‘logloss’, n_estimators = 50, objective = ‘binary:logistic’, use_label_encoder = False
LGBM	0.87	13	colsample_bytree = 1, learning_rate = 0.1, max_depth = -1, min_child_weight = 0.001, min_split_gain = 0, n_estimators = 100, num_leaves = 20, reg_alpha = 0, reg_lambda = 0

The row highlighted in gray indicates the most effective classification method using the FISHER selection method.

**Table 6:** Best classification models. The letter “E” appearing in the model symbol denotes the embedded method.

Model Symbol	Feature Selection Method	Classification Method	The Number of Features
F-RF-21	FISHER	RF	21
A-RF-12	ANOVA	RF	12
R-RF-23	RELIEF	RF	23
SFS-RF-21	SFS	RF	21
SBS-RF-22	SBS	RF	22
RFE-RF-15	RFE	RF	15
E-LR-3	E	LR	3
E-ADA-4	E	ADABoost	4
E-LGBM-23	E	LGBM	23

**Table 7:** Model evaluation results.

Model Symbol	Class	PRE	REC	F1	TPR	TNR	ACC
F-RF-21	Non-COVID-19	0.84	0.88	0.86	0.83	0.88	0.85
	COVID-19	0.86	0.83	0.84			
A-RF-12	Non-COVID-19	0.80	0.83	0.82	0.78	0.83	0.81
	COVID-19	0.82	0.78	0.80			
R-RF-23	Non-COVID-19	0.84	0.88	0.86	0.83	0.88	0.85
	COVID-19	0.86	0.83	0.84			
SFS-RF-21	Non-COVID-19	0.81	0.88	0.84	0.78	0.88	0.83
	COVID-19	0.86	0.78	0.82			
SBS-RF-22	Non-COVID-19	0.84	0.88	0.86	0.83	0.88	0.85
	COVID-19	0.86	0.83	0.84			
RFE-RF-15	Non-COVID-19	0.84	0.88	0.86	0.83	0.88	0.85
	COVID-19	0.86	0.83	0.84			
E-LR-3	Non-COVID-19	0.79	0.92	0.85	0.74	0.92	0.83
	COVID-19	0.89	0.74	0.81			
E-ADA-4	Non-COVID-19	0.83	0.83	0.83	0.83	0.83	0.83
	COVID-19	0.83	0.83	0.83			
E-LGBM-23	Non-COVID-19	0.78	0.88	0.82	0.74	0.88	0.81
	COVID-19	0.85	0.74	0.79			

**Table 8:** The full set of features used to build the RFE-RF-15 model. The order of the features follows their ranking by the recursive feature elimination method.

No.	Feature Symbol	Feature Name	COVID-19 Cases, n = 77	Non-COVID-19 Cases, n = 51
			Number of Patients (%) or Mean (SD)	Number of Patients (%) or Mean (SD)
1	X2	Age (years)	63.9 (10.1)	44.3 (12.8)
2	X3	Shortness of breath at rest	71 (92.2)	51 (100)
3	X70	Leukocytes	5.1 (2.6)	4.5 (1.7)
4	X72	Granulocytes	5.4 (3.4)	6.1 (5.2)
5	X9	COVID test	30 (39.0)	23 (45)
6	X37	COPD	12 (15.6)	4 (8)
7	X4	Shortness of breath during exercise	61 (79.2)	45 (88)
8	X71	Potassium (K)	3.99 (0.7)	4.2 (0.9)
9	X31	Diabetes mellitus	12 (15.6)	0 (0)
10	X10	Diarrhea	2 (2.6)	4 (8)
11	X53	Dry rhonchi	15 (19.5)	11 (22)
12	X13	Whistles in the chest	4 (5.2)	3 (6)
13	X68	Hemoglobin	132.33 (16.3)	129.5 (12.7)
14	X29	Stenting	5 (6.5)	1 (2)
15	X135	Medical history number	1105.9 (731.5)	980.2 (744.9)

**Table 9:** The full set of features used to build the E-ADA-4 model. The order of the features follows their ranking by the SelectFromModel method with AdaBoost estimator.

No.	Feature Symbol	Feature Name	COVID-19 Cases, n = 77	Non-COVID-19 Cases, n = 51
			Number of Patients (%) or Mean (SD)	Number of Patients (%) or Mean (SD)
1	X135	Medical history number	1105.9 (731.5)	980.2 (744.9)
2	X2	Age	63.9 (10.1)	44.3 (12.8)
3	X56	Respiratory rate	20.1 (4.1)	20.1 (2.1)
4	X68	Hemoglobin	132.33 (16.3)	129.5 (12.7)

**Table 10:** Summary of results of similar tests.

No. in Refs.	Material	Method	Results
Authors’ research	Comprehensive set of diagnostic information (CSDI) including, among other things, medical history, demographic data, signs and symptoms of the disease, and laboratory results	Random forest	Accuracy: 85%, sensitivity: 83% specificity: 88%.
[36]	Combination of chest X-ray and CT images	VGG19+CNN model	Accuracy: 98.05%, recall: 98.05%, precision: 98.43%, specificity: 99.5%.
[35]	CT chest images: 90 COVID-19 patients; 90 other pneumonias patients	SVM	Accuracy: 89.83%, sensitivity: 94.22%, specificity: 85.44%, AUC: 0.940.
[41]	Vibrational spectroscopy: the original saliva FTIR spectra	Grey wolf optimized support vector machine (GWO-SVM)	Accuracy: 98.25%, specificity: 97.14%, F1 score: 97.78%.
[13]	Blood tests	Random forest	Accuracy: 92%, sensitivity: 71%, specificity: 96%, AUC: 91%.
[42]	Blood gas parameters	CHAID decision tree	Accuracy: 68.2%
[43]	Biochemical, hematological, and immunological blood tests	LogNNet 51:50:20:2 architecture	Accuracy: 90.9%, precision: 99.0%, recall: 91.4%.
[44]	Routine blood tests	Deep learning (DL), K-nearest neighbors (KNN), and support vector machines (SVM)	Accuracy: 86%, sensitivity: 95%.
[45]	Laboratory features	SVM	Accuracy: 77.5%, specificity: 78.4%.
[46]	Laboratory features	XGBoost classifier	Accuracy: 75%, specificity: 49%.
[47]	Laboratories and clinical features	SVM, RBF, LR, KNN	AUC 83.4%
[20]	Clinical data	Naive Bayes, Bayesian network, random forest (RF), multilayer perceptron, K-star, C4.5, and support vector machine	Accuracy: 92.42%, specificity: 75.70%, precision: 92.30%, sensitivity: 92.40%, F-measure: 92.00%, ROC: 97.15%.
[48]	Fourteen clinical features	BayesNet, logistic, IBk, CR, PART, and J48	Accuracy: 84.21%
[49]	Datasets containing demographic information; related tests, including symptoms, vital signs, lab results, values from routine blood tests	Improved binary flamingo search algorithm (IBFSA) + SVM	Accuracy: 95.05%, precision: 94.13%, recall: 98.16%, F-measure: 96.09%.
[40]	Hexaxial feature mapping represent 12-lead ECG to 2D colorful images	CNN: ResNet50	Accuracy: 96.20%, F1 Score: 96.30%.

## Data Availability

Not applicable.
